# Increasing Physical Activity With Mobile Devices: A Meta-Analysis

**DOI:** 10.2196/jmir.2171

**Published:** 2012-11-21

**Authors:** Jason Fanning, Sean P Mullen, Edward McAuley

**Affiliations:** ^1^Department of Kinesiology and Community HealthUniversity of Illinois at Urbana-ChampaignUrbana, ILUnited States

**Keywords:** Behavior change, exercise, meta-analysis, mobile phone, physical activity, review

## Abstract

**Background:**

Regular physical activity has established physical and mental health benefits; however, merely one quarter of the U.S. adult population meets national physical activity recommendations. In an effort to engage individuals who do not meet these guidelines, researchers have utilized popular emerging technologies, including mobile devices (ie, personal digital assistants [PDAs], mobile phones). This study is the first to synthesize current research focused on the use of mobile devices for increasing physical activity.

**Objective:**

To conduct a meta-analysis of research utilizing mobile devices to influence physical activity behavior. The aims of this review were to: (1) examine the efficacy of mobile devices in the physical activity setting, (2) explore and discuss implementation of device features across studies, and (3) make recommendations for future intervention development.

**Methods:**

We searched electronic databases (PubMed, PsychINFO, SCOPUS) and identified publications through reference lists and requests to experts in the field of mobile health. Studies were included that provided original data and aimed to influence physical activity through dissemination or collection of intervention materials with a mobile device. Data were extracted to calculate effect sizes for individual studies, as were study descriptives. A random effects meta-analysis was conducted using the Comprehensive Meta-Analysis software suite. Study quality was assessed using the quality of execution portion of the *Guide to Community Preventative Services* data extraction form.

**Results:**

Four studies were of “good” quality and seven of “fair” quality. In total, 1351 individuals participated in 11 unique studies from which 18 effects were extracted and synthesized, yielding an overall weight mean effect size of *g* = 0.54 (95% CI = 0.17 to 0.91, *P* = .01).

**Conclusions:**

Research utilizing mobile devices is gaining in popularity, and this study suggests that this platform is an effective means for influencing physical activity behavior. Our focus must be on the best possible use of these tools to measure and understand behavior. Therefore, theoretically grounded behavior change interventions that recognize and act on the potential of smartphone technology could provide investigators with an effective tool for increasing physical activity.

## Introduction

It is well documented that regular physical activity is associated with reduced morbidity and mortality attributable to such diseases as cardiovascular disease, diabetes, and some cancers [1-4]. Unfortunately, participation rates have remained dismally low in spite of significant scientific endeavors to enhance participation, reduce attrition, and increase maintenance of this important health behavior. In 2007, less than half of all adults in the United States achieved recommended levels of physical activity [5]. Furthermore, past research suggests that among those beginning a new exercise program, 50% will drop out within six months [6]. The advent of new and ubiquitous technologies offers a potential solution to increasing the effectiveness of and adherence to physical activity interventions. One such technology is the mobile telephone, which has shown promise as a delivery mechanism for health behavior interventions. These devices have become a more pervasive part of society with usage rates increasing exponentially. For example, in the United States some 83% of adults own mobile phones, while in younger populations (ie, 18-24 year olds) as many as 95% own mobile phones [7]. In the United States [8], Australia [9], and Western Europe [10], activated mobile phones outnumber citizens.

Declining cost and enhanced versatility in features are likely contributors to the rapid increase in mobile phone usage, and short message service (SMS, ie, text-messaging) has become an almost universal way to engage in brief conversations and convey short messages. In the United States, 73% of all adult cell phone users send daily text messages, at an average rate of 39.1 per day. Among younger Americans, a staggering 97% of mobile phone users send text messages at an average rate of 87.7 messages per day [7]. In total, nearly 2.1 trillion messages were sent in 2010 [8]. In the late 2000s, the growth of feature phones (ie, devices capable of basic voice and multimedia functions) was supplanted by surging growth in the smartphone market. These devices originally combined the computing power of handheld computers with mobile communication features serving primarily adult professionals. As of May 2011, 35% of all mobile consumers in the U.S. owned smartphones, and rates were higher in ethnic minorities [7]. Smartphones are equipped with advanced technological features that distinguish them from the feature phone. Typically they are capable of sending and receiving information via the Internet, connecting to local wireless networks and Bluetooth devices, utilizing global positioning system (GPS) data and allowing users to download countless mobile applications straight to their device from just about any location.

Behavioral scientists have begun to realize the potential of mobile devices to understand multiple health behaviors, and meta-analyses have supported the efficacy of mobile technology for influencing behaviors including diabetes management [11] and smoking cessation [12]. It is clear that some of the unique qualities of these devices would be attractive features for physical activity interventions allowing scientists to: collect objective and self-report measures of activity in real time; provide feedback and support at the point of decision; provide interactive, immersive, and individualized content that is automatically generated; and deliver materials on a device that is already carried by the individual [13]. A number of reviews exist examining the use of technologies that offer similar benefits to mobile devices and their effects on health behaviors. For example, Goode et al [14] reviewed telephone-based interventions for influencing physical activity and dietary behaviors. Multiple researchers have examined aspects of Internet-delivered interventions [15,16]. Others have examined the use of mobile technology to aid in disease prevention and management [17], as well as the influence of SMS technology on various health behaviors (eg, smoking cessation, diabetes self-management, asthma self-management; [18]). To date, however, no meta-analysis has been conducted that examines the efficacy of mobile devices for changing physical activity behavior. Given the increase in research applications of such devices, we believe that a comprehensive analysis of the influence of mobile devices on physical activity behavior would afford future researchers a foundation for guiding subsequent interventions. Herein, we present a meta-analysis of interventions that have utilized mobile devices (ie, PDA or mobile telephones) to influence physical activity behavior.

## Methods

### Search Strategy

An extensive search of online electronic databases (PsychINFO, PubMed, Scopus) was conducted between August 15, 2011, and July 3, 2012, in which we sought articles published since the year 2000. The following search string was utilized across the three databases: ((mobile phone) OR (cell phone) OR PDA OR SMS OR (text message)) AND ((physical activity) OR exercise).

### Inclusion and Exclusion Criteria

For inclusion in this analysis, studies were required to be published or in press, in the English language, and to incorporate mobile technologies in the collection or dissemination of intervention materials meant to positively influence physical activity behavior. This included data collection or conveyance of intervention information via SMS, as well as implementation of native mobile device software or hardware. Studies were required to include a comparison group and to provide original data sufficient for calculating Cohen’s *d* effect sizes (ie, baseline and follow-up means and baseline standard deviation). These criteria are intentionally broad, as relatively few relevant studies have been published.

Articles that described proof-of-concept trials, conference proceedings, or review articles were excluded from this analysis. Outcomes from studies that were not explicitly related to physical activity were also excluded, as were studies in which participants interacted with the mobile component less than one time per week.

### Review Procedure

**Figure 1:**
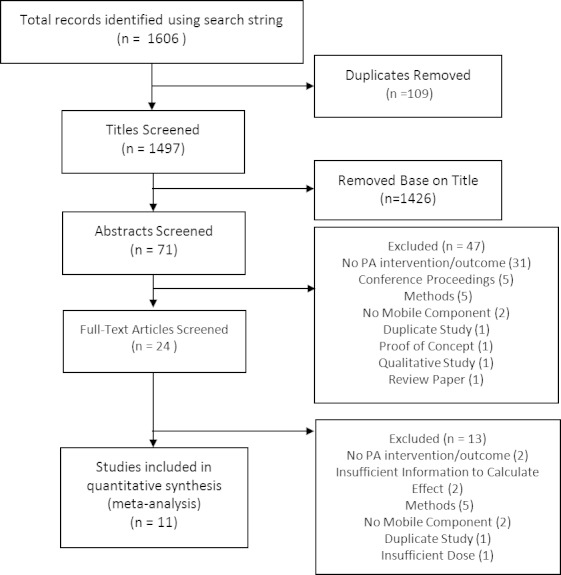
Flow diagram of study selection.

Study selection was conducted in four phases (see Figure 1). During the initial stage, all citations from each database query were imported into a central citation manager (EndNote X5 [19]), which facilitated removal of duplicates. Next, the first two authors searched titles for publications that referenced physical activity and a mobile device, removing those that definitively did not match inclusionary criteria. In the following stage, both reviewers examined abstracts of the remaining articles, further screening out articles that did not meet criteria. During the final stage, full-text citations were reviewed to make sure that all criteria were met, and study descriptives were extracted and tabulated. When study length was reported in months rather than weeks, a four-week month was assumed. Following this review, search results were compared between the two authors, with the third author acting as arbiter to any inconsistencies. When physical activity outcomes were reported but were not sufficient to calculate effect sizes, study authors were contacted to determine means and standard deviations at all time-points (n = 7). In the event that this information could not be obtained, the effect was excluded from analysis (n = 3 [20-22]). Reference lists of included articles and relevant reviews were searched for additional articles, and direct requests were made to experts in the area of mobile health for additional studies in review or in press.

### Study Quality Assessment

In order to assess quality at the study level, the quality of execution portion of the *Guide to Community Preventative Services* data extraction form was used [23]. This form addresses six categories of threats to study validity (ie, population and study description, sampling, measurement, data analysis, interpretation, and other limitations). There are nine possible limitations across these six categories. Studies with 0-1 limitations are deemed to be of “good” quality, those with 2-4 limitations are of “fair” quality, and those with more than four limitations are deemed to be of “poor” quality. This assessment is meant for descriptive purposes only, and as such no studies were excluded due to their quality rating.

### Synthesis of Results

For each study we computed standardized mean differences (Cohen’s *d*) by subtracting the mean change in the control group from the mean change in the treatment group. Pre- and post-intervention means were used to calculate mean change in order to remain consistent across all studies. This was divided by the pooled baseline standard deviation [24]. Baseline sample size was utilized in calculating pooled standard deviation. For multiple group versus control comparisons, pooled means and standard deviations were calculated in accordance with the *Cochrane Handbook for Systematic Reviews of Interventions* [25].

After obtaining standardized mean differences, we conducted a random-effects meta-analysis, using the Comprehensive Meta-Analysis [26] software suite. Random-effects meta-analyses account for heterogeneity of included measures, if they are indeed related (ie, they measure physical activity [27]). From the software, we obtained Hedge’s *g* for the overall effect, which is less biased for small sample sizes, as well as for the duration of moderate to vigorous physical activity duration (MVPA duration) and steps, as these were the only outcome measures reported across multiple studies. Finally, we calculated the effect for studies that utilized mobile phones as well as those that distributed materials on PDA devices. We also obtained a heterogeneity statistic (Q) for each of these effects, which we used to calculate *I* ^2^. This allows one to examine the percentage of observed variance that is real, with low percentages indicating that most variance is spurious (pp.117-119) [28].

## Results

### Study Selection

Our initial search yielded 1606 publications, of which 109 were removed as duplicates. From the remaining 1497 titles, 1426 were removed based on title. Subsequently, 47 of 71 remaining publications were removed after abstracts were screened, and an additional 13 were removed upon review of the full text article. Reasons for removal are detailed in Figure 1. We contacted 7 authors in order to obtain data sufficient to calculate effect sizes. Of these, 2 could not be reached, therefore those publications were removed.

### Study Quality

Four studies were classified to be of “good” quality [29-32], and seven studies were classified to be of “fair” quality [33-39]. The most frequently violated items from the data extraction form were: “Was the population that served as the unit of analysis the entire eligible population or a probability sample at the point of observation?” [33,34,36-38], “Was there an attempt to measure exposure to the intervention?” [32,33,37-39], and “Did the authors control for differential exposure to the intervention?” [30,31,34,37,38].

### Intervention Characteristics

Eleven unique studies were included in this analysis (n = 1351). Of these, eight reported use of SMS [31-35,37-39], four reported use of native mobile software [29,30,36,37], and two reported use of a personal digital assistant (PDA) rather than a mobile phone [29,30]. Several studies were classified in more than one category (eg, mobile phone & SMS). Five studies reported duration of moderate to vigorous physical activity (MVPA duration) [30,31,34,35,39], three reported pedometer step counts [33,36,37], one reported frequency of MVPA (MVPA frequency) [34], another reported percent of active time spent in MVPA (% MVPA) [37], one study reported accelerometer counts per minute [31], two reported metabolic equivalents (METs) [29,32], and one reported number of days per week of walking for exercise as well as number of days exercising per week [38]. Intervention duration ranged from 2 to 52 weeks and averaged 14.6 weeks. Sample sizes ranged from 17 to 357 participants (*M* = 121.1 participants), and mean participant ages ranged from 8.7 to 68 years. Detailed characteristics of all studies included in the meta-analysis can be found in Table 1.

### Data Synthesis

From the 11 included studies, 18 effects were extracted and synthesized. From these effects, the random-effects meta-analysis yielded a significant moderate overall weighted mean effect size of *g =* 0.54 (95% CI = 0.17 to 0.91, *P* = .005). The heterogeneity within these studies was significant (*Q* = 87.79, df = 10, *P* < .001, *I* ^*2*^ = 88.61%), supporting the use of random effects meta-analysis.

**Table 1:** Intervention characteristics.

Study authors	n	Mobile component	Utilization	Length (weeks)	Age M (SD)
Cheung, Chow, & Parfitt (2008) [33]	52	SMS^a^	Relay information about PA benefits	6	INT^b^: 38.9 (10.8) CON^c^: 26.5 (1.9)
Conroy et al (2011) [29]	210	PDA^d^ & PDA + feedback (FB)	Tailored, automated FB	~24	47.3 (8.8)
Fjeldsoe, Miller, & Marshal (2010) [34]	88	SMS	Tailored SMS. SMS also sent to dedicated social support individual	12	30 (6)
Hurling et al (2007) [35]	77	SMS	Relay reminders & motivational messages	9	40.4 (7.6)
King et al (2008) [30]	37	PDA	PDA self-monitoring, weekly FB, goal setting, support	8	60.2 (7.1)
Kirwan et al (2012) [36]	200	Smartphone App	Self-monitoring of steps using the mobile app and/or the intervention website	12	39.9 (12.3)
Lubens et al (2012)[31]	357	SMS	Relay social support	52	13.8 (0.45)
Nguyen et al (2009) [37]	17	Native App & SMS	Mobile self-monitoring with tailored SMS feedback	~24	68 (11)
Prestwich, Perugini, & Hurling (2010) [38]	134	SMS	Relay implementation intention or goal reminders	4	23.4 (5.6)
Shapiro et al (2008) [39]	40	SMS	Daily self-monitoring messages with automated, tailored feedback	8	8.7 (2.3)
Sirriyeh, Lawton, & Ward (2010) [32]	120	SMS	Relay affective messages, instrumental messages, or combined messages	2	17.3 (.7)

^a^ SMS: Short Message Service.

^b^ INT: Intervention group.

^c^ CON: Control group.

^d^ PDA: Personal digital assistant.

**Figure 2:**
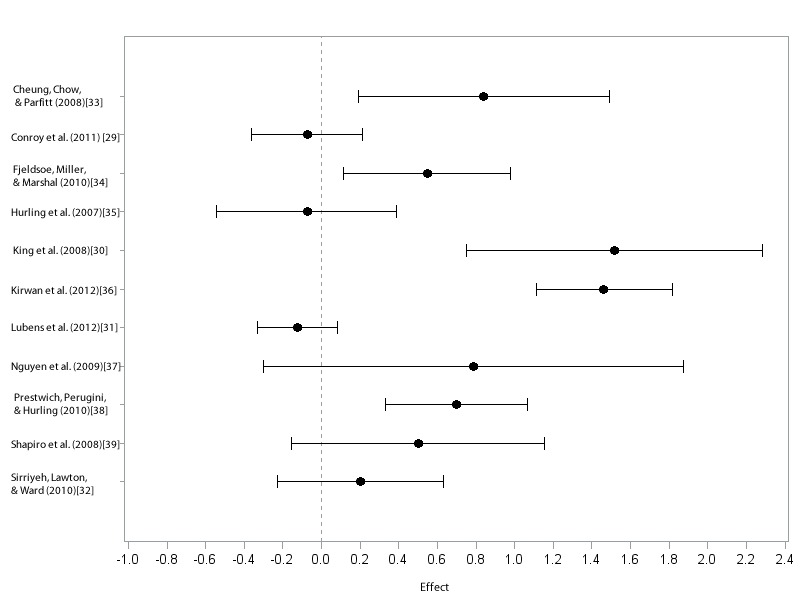
Forest plot of individual effect sizes (d).

Although there are insufficient numbers of studies to reliably examine between outcomes differences, for illustrative purposes, we examined MVPA duration (five studies) and pedometer steps (three studies) independently, as they were the most frequently reported outcomes. Effects for each study can be found in Table 2 and are also displayed in Figure 2.

**Table 2:** Study outcomes.

Study authors	Quality	Outcomes	INT^a^ M Change (Baseline SD)	CON^b^ M Change (Baseline SD)	d
Cheung, Chow, & Parfitt (2008) [33]	“Fair”	Steps to work	-1.5 (14)	1.2 (18.7)	-0.18
		Steps at work	1.3 (2.5)	-1.8 (2.5)	1.26
		Steps off work	4 (6.6)	0.4 (8.4)	0.52
Conroy et al (2011) [29]	“Good”	MET^c^-hours (combined PDA^d^)	6.31 (17.87)	7.57 (15.17)	-0.08
Fjeldsoe, Miller, & Marshal (2010) [34]	“Fair”	MVPA^e^ frequency	1.82 (1.48)	0.24 (1.44)	1.09
		MVPA duration	18.26 (170.46)	16.36 (170.49)	0.01
Hurling et al (2007) [35]	“Fair”	Accelerometer counts spent in MVPA	-9.5 (52.1)	-5.5 (53.1)	-0.08
King et al (2008) [30]	“Good”	MVPA duration	177.7 (114.5)	-80 (215)	1.55
Kirwan et al (2012) [36]	“Fair”	Total days logged	22.76 (12.8)	1.26 (12.1)	1.76
		Steps	159.89 (3308.36)	-4360.7 (3987.2)	1.19
Lubens et al (2012) [31]	“Good”	Accelerometer counts/min	-90.9 (420)	-43.7 (395.4)	-0.12
		MVPA duration	-13.1 (36.8)	-8.3 (35.1)	-0.13
Nguyen et al (2009) [37]	“Fair”	Steps	609 (3020.76)	-1017 (3021)	0.57
		% MVPA	4.4 (7.64)	-3.5 (7.8)	1.09
Prestwich, Perugini, & Hurling (2010) [38]	“Fair”	Days/wk walking > 30 min	1.4 (1.19)	0.47 (1.17)	0.79
		Days/wk exercising > 30 min	1.85 (1.48)	0.94 (1.52)	0.62
Shapiro et al (2008) [39]	“Fair”	MVPA duration	34.4 (48.5)	-15.1 (126.3	0.51
Sirriyeh, Lawton, & Ward (2010) [32]	“Good”	MET-minutes	3145.26 (11681.71)	819.45 (11347.71)	0.20

^a^ INT: Intervention group.

^b^ CON: Control group.

^c^ MET: Metabolic equivalents of task.

^d^ PDA: Personal digital assistant.

^e^ MVPA: Moderate to vigorous physical activity.

There was significant moderate to large effect for pedometer steps (*g =* 1.05, 95% CI = 0.75 to 1.35, *P* < .01). When examining intervention components specifically, those delivered via mobile phone yielded a significant moderate effect (*g =*.52, 95% CI = 0.11 to .94, *P* = .01). The effects were non-significant for both MVPA duration (*g =* 0.20, 95% CI = -0.19 to 0.60, *P =* .31) as well as for PDA delivered (*g* = .68, 95% CI = -0.88 to 2.25, *P* = .39), with lacking significance in the latter likely due in large part to the small number of studies and considerable heterogeneity.

## Discussion

Previous reviews and meta-analyses have identified the mobile platform as an effective means to influence multiple health behaviors, including diabetes management [11] and smoking cessation [12]. The present meta-analysis provides some preliminary support for interventions using mobile technology to increase physical activity behavior. Although the use of mobile technology in physical activity research is still in its infancy, we believe that this finding serves as an important foundation for informing the development of appropriate and efficient intervention techniques using such technology to enhance this important health behavior.

However, this initial enthusiasm must be tempered by consideration of the limitations inherent in the individual studies reviewed. First, because of its broad accessibility during most of the included interventions, SMS is the primary technology utilized in this review, which hampers our ability to make evidence-based statements regarding the efficacy of interventions that utilize smartphones. Further, one primary weakness in the studies reviewed here is the inability to determine the unique contribution of the mobile device component to changing physical activity behavior. For the most part, mobile devices have been used primarily as data collection methods (eg, steps reported via SMS) or as supplemental materials (eg, provision of feedback via SMS) to a broader behavior change intervention relying on more traditional methods (eg, face-to-face counseling). When incorporated into larger interventions, SMS messages present a nimble and efficient means to relay feedback and information to participants, provide participants freedom in accessing the intervention message, and to an extent, SMS allows for real-time assessment of behavior.

For example, Fjeldsoe et al [34] developed an SMS-based intervention to increase physical activity in post-natal mothers who were sent bi-weekly, social cognitive-based motivational messages tailored to the study participants. In addition to these messages, a goal-setting refrigerator magnet and face-to-face and telephone counseling were provided at baseline, and telephone counseling alone was provided at week six. The researchers created a useful and innovative model of population-specific text-messaging; however, the inclusion of the counseling and magnet components highlights the inability of current SMS-based interventions to be all-encompassing. This is in contrast to the current movement by many in the area of Internet-based health research who recommend full automation (ie, minimal researcher contact, few supplementary materials, and automatic generation of individualized user content). Hurling et al [35] used an automated design in creating an Internet-based physical activity intervention that utilized SMS to deliver motivational and reminder messages to participants. The intervention was effective; however, our understanding of the efficacy of the mobile component is again clouded by its role as an SMS supplement to the broader, Internet-based trial.

Unfortunately, the simple adoption of mobile technologies does not ensure effective intervention methods. Theoretical frameworks should guide interventions to help researchers understand which components were most effective in successful (or unsuccessful) trials. For example, Prestwich et al [38] instructed participants to develop implementation intentions and a goal relative to brisk walking. These intentions are based in part on Gollwitzer’s [40] position that anticipating and planning goal-directed responses removes some burden of responsibility from the individual and delegates it to the situation. That is, implementation intentions are formed in such a way that one self-regulates in a manner following “when I encounter X, I will respond by Y” [40], thus ensuring that when a particular situation is encountered, the desired response occurs automatically. After forming goals and implementation intentions, subjects were assigned to one of three groups: one that received SMS reminders of their implementation intentions, one that received SMS reminders of their brisk walking goal, and a control group. Both SMS groups significantly increased the amount of walking relative to control. Further, the SMS plus goal group better recalled their goals, while the SMS plus implementation intentions group better recalled their implementation intentions, indicating that this form of mobile intervention successfully supported the underlying theoretical principles of the study.

It is worth considering, however, that much can be added to current theoretical models of behavior change so that they are better suited to design mobile interventions and interpret results. Ritterband et al [41] have developed a behavior change model for Internet-based interventions that can be used to guide mobile interventions, given the similarities of the two platforms. The authors note that at the time of writing, no theoretical model existed to guide development and testing of Internet-based interventions. More recently, Riley and colleagues [13] questioned whether or not current behavioral theories are adequate for designing and implementing mobile interventions. They acknowledge that current models account for the state of the participant at baseline and challenge that they do not adequately account for the interplay between user experiences and the dynamic and adaptable nature of mobile interventions. For Internet and mobile interventions alike, theories that account for inter- and intra-individual change over time may be a better fit.

One of the unique and advantageous features of mobile devices, and smartphones in particular, is their use of the popular and widespread native applications or “apps”. Rather than relying on Internet connectivity to deliver content that resides on a remote server (ie, the method utilized by “web apps”), native apps are developed for the mobile operating system, reside on the user’s mobile device, and may store data locally or exchange it via the Internet. Importantly, native apps allow a greater degree of flexibility and complexity to software and intervention designers. Since 2008, application development has continued to grow across multiple platforms at an exponential rate and has mirrored the increase in smartphone users worldwide [42].

Though limited in number at the time of writing, research using native applications gives cause for optimism. For example, King et al [30] developed software that cued individuals to participate in a short survey twice daily. This survey assessed types of physical activity, context of physical activity, and behavioral/motivational factors. The physical activity program was grounded in social cognitive principles and included an assessment of barriers and enablers; self-regulation of step counts; and a goal-setting component, from which personalized PDA-delivered graphical/textual feedback was developed. The authors reported a mean increase in moderate to vigorous physical activity of 177.7 minutes per week, as compared with a mean decrease of 80 minutes in the control group over eight weeks. This early native application−based intervention demonstrated the versatility and potential efficacy of this mode of delivery.

Finally, inclusion of advanced sensors (eg, integrated accelerometer and GPS devices) holds promise for more accurate assessment of physical activity behavior in real time. Ecological Momentary Assessment (EMA) is a measurement strategy that aims to collect data reflecting behavior and the social/physical context that influences such behavior. Often this is accomplished by providing a prompt to the participant, cuing them to submit reports relative to, for example, their current location, the type of activity they are currently participating in, and their social context [43-45]. In combination with smartphone technology, integrated motion sensing can bolster the accuracy of activity measurement, while GPS data may provide geographic location information, allowing for a more detailed examination of the environmental context in which activity does or does not occur [44]. Bergman et al [46] have questioned the validity of mobile phone−based accelerometry, although their study examined only a single mobile application meant to estimate number of steps taken. Recently, Wu et al [47] used accelerometer and gyroscope data obtained by smartphone devices to classify activity types using machine learning algorithms. They found that they could accurately classify walking and jogging activities with greater than 90% accuracy. However, there still remain issues to resolve relative to the best location on the body to obtain accurate movement data on larger and more diverse populations, and the most accurate algorithms for quantifying different types and intensities of activity.

### Strengths and Limitations

A primary strength of this study is its status as the first meta-analysis examining the influence of mobile devices on physical activity behavior. Acknowledging the efficacy of current interventions while addressing advances in technology can help to guide future intervention development. We must also note several limitations inherent in this meta-analysis. First, the small number of published studies necessitated broad inclusionary criteria, thereby including studies that varied greatly in population characteristics, study design, and use of mobile components. Further, study heterogeneity, as denoted by the *I* ^2^ statistic, should be interpreted with caution, particularly due to the small number of effects included. However, aggregation of study effects and designs is important in order to effectively utilize and improve on current designs.

### Conclusion

Given that smartphones only recently acquired enough market penetration to warrant implementation as a health behavior change platform, it is not surprising that there has been little rigorous study of the influence of this technology on physical activity. Fortunately, innovative research using SMS, PDAs, and the Internet has laid a foundation on which smartphone research can be built. As the field increasingly utilizes this novel technology, our focus must not be on any one specific device but on the best possible use of these tools to measure and understand behavior. Indeed, scientifically rigorous, theoretically grounded behavior change interventions that recognize and act on the potential of smartphone technology (eg, integrated accelerometry, Internet connectivity, ubiquitous presence) could provide investigators with an efficient and effective tool and participants with an immersive and exciting experience.

## Abbreviations

CI: Confidence Interval
CON: Control Group
EMA: Ecological Momentary Assessment
FB: Feedback
GPS: Geographic Positioning System
II: Implementation Intentions
INT: Intervention Group
MET: Metabolic Equivalent of Task
MVPA: Moderate to Vigorous Physical Activity
PDA: Personal Digital Assistant
SMS: Short Message Service (ie, text messaging)
